# Modafinil’s effects on cognition and sleep quality in affectively-stable patients with bipolar disorder: a pilot study

**DOI:** 10.3389/fpsyt.2023.1246149

**Published:** 2023-09-04

**Authors:** Jessica M. Lipschitz, Mercedes Perez-Rodriguez, Marzieh Majd, Emmett Larsen, Joseph Locascio, Chelsea K. Pike, Megan Shanahan, Katherine E. Burdick

**Affiliations:** ^1^Department of Psychiatry, Brigham and Women’s Hospital, Boston, MA, United States; ^2^Department of Psychiatry, Harvard Medical School, Boston, MA, United States; ^3^Department of Psychiatry, Icahn School of Medicine at Mount Sinai, New York, NY, United States; ^4^Department of Psychology, Stony Brook University, Stony Brook, NY, United States; ^5^Department of Neurology, Massachusetts General Hospital, Boston, MA, United States; ^6^Department of Neurology and Harvard Catalyst Biostatistical Group, Harvard Medical School, Boston, MA, United States

**Keywords:** bipolar disorder, modafinil, cognitive functioning, sleep, daytime sleepiness

## Abstract

**Introduction:**

Despite advances in the treatment of bipolar disorder (BD), most patients do not achieve complete inter-episode recovery and functional disability is common. During periods of relative remission, many patients continue to experience neurocognitive dysfunction, reduced daytime activity levels, and sleep disturbances. This 8-week, randomized, placebo-controlled pilot study evaluated the feasibility, safety and preliminary efficacy of the wake-promoting drug, modafinil (Provigil^®^), on neurocognitive functioning, daytime sleepiness, and sleep quality in affectively-stable BD patients.

**Methods:**

Twelve individuals with affectively-stable BD were recruited and randomized to a flexible dose of modafinil (100 to 200 mg/day) or placebo, adjunctive to a therapeutic dose of a mood stabilizer. Weekly in-person visits tracked sleep quality and daytime sleepiness as well as side effects and mood symptoms. Neurocognitive functioning was assessed at baseline, week 4, and week 8.

**Results:**

No serious adverse events were reported. Newly emergent side effects in the modafinil group included heart palpitations, itching, fatigue, and decreased energy. Two patients discontinued modafinil owing to side effects and one of these patients withdrew from the study. One patient discontinued placebo and was withdrawn from the study. Preliminary evaluations of clinical efficacy showed a marginally significant interaction between treatment group and time in two cognitive domains (speed of processing and verbal learning), indicating greater improvement in the modafinil group versus placebo. Additionally, there was a marginally significant effect of treatment group on daytime sleepiness, suggesting lower daytime sleepiness in the modafinil group versus placebo. Counterintuitively, we found a significant treatment group by time interaction effect on sleep quality, suggesting greater improvement in sleep quality in the placebo group versus the modafinil group.

**Discussion:**

Results suggest that modafinil is a relatively safe medication for affectively-stable BD patients when given with adjunctive mood stabilizers. Results are suggestive of cognitive benefit and improved daytime sleepiness, but worse sleep quality in those patients prescribed modafinil. A fully powered clinical trial is warranted with specific attention to the characteristics of patients who are most likely to benefit from treatment with modafinil and other methodological lessons learned from this pilot.

**Clinical trial registration:**

ClinicalTrials.gov, identifier NCT01965925.

## 1. Introduction

Complete symptomatic remission and functional recovery are not the norm during inter-episode recovery periods in BD patients (1–3). The presence of continuing symptoms has a profound impact with a direct influence on clinical course and functional outcome (4, 5). Cognitive impairment and circadian dysfunction persist in the majority of BD patients even when acute symptoms remit. As many as 70% of euthymic BD patients have shown diminished sleep efficiency and decreased daytime activity levels in addition to deficits in the domains of attention, verbal memory, and executive functioning (6, 7). While these persistent problems contribute directly to functional impairment and reduced quality of life, they are not yet a primary focus of treatment. If patients are to achieve a more complete inter-episode recovery, these aspects of the illness warrant directed intervention.

The nature of the circadian abnormality in BD is not known, however recent work proposes detachment of the biological clock from environmental variables that regulate circadian rhythms, and/or an out of phase sleep–wake cycle (8). The relationship between sleep quality, daytime wakefulness, and neurocognition seems intuitive with sleep deprivation resulting in lower energy and impaired cognition in animals and humans (9).

A majority of BD patients demonstrate deficits in attention, memory, and executive functioning even when affectively-stable (7). Although several features of the illness potentially contribute to the persistent cognitive impairment noted during euthymic periods, the circadian-based deficits in sleep quality and daytime wakefulness are likely to exacerbate cognitive problems in BD (10), as has been shown in healthy controls, sleep disordered subjects, and other clinical conditions including BD (11, 12).

When considering agents that may simultaneously improve upon sleep quality and enhance cognition, the wake-promoting agent, modafinil, is an ideal candidate. It is FDA-approved for improving wakefulness in adults with excessive daytime sleepiness due to primary sleep disorders (Provigil^®^, 2007). Additionally, while it is characterized as a psychostimulant, it has been favorably characterized with regard to side effects and abuse/dependence potential in comparison with amphetamine (13), making it a safer option for use in BD, an illness with very high comorbidity for substance abuse (14). Modafinil and armodafinil have been found to be safe and effective in treating depression in BD, when given adjunctive to a mood stabilizing agent (15). Finally, modafinil exhibits robust effects on various neurotransmitter systems in the brain, including catecholamines, serotonin, glutamate, gamma-aminobutyric acid (GABA), orexin, and histamine (16). As a result of these neurochemical actions, modafinil holds significant promise as a potential treatment for cognitive dysfunction in neuropsychiatric disorders. Modafinil has been shown to enhance cognition in healthy controls, sleep-disordered individuals, neurological patients, and patients with schizophrenia and major depressive disorder (16, 17), but its effects on cognition in BD are not yet known.

We completed an 8-week, placebo-controlled, pilot study of modafinil in a small sample of affectively-stable patients with BD. The primary goal of this pilot study was to assess the safety and feasibility of adjunctive modafinil in affectively-stable outpatients with BD. The focus on sleep quality and daytime sleepiness as key outcomes was directly related to the primary mechanism of action of modafinil in “promoting wakefulness” – an effect that we believed would have downstream effects in BD patients on cognition. As a secondary aim, though underpowered for inferential statistics, we investigated whether modafinil shows a positive signal for improving sleep quality, daytime wakefulness, and cognitive functioning in BD.

## 2. Materials and methods

This study was reviewed and approved by the Institutional Review Board (IRB) at the Icahn School of Medicine at Mount Sinai. All patients signed an informed consent document before any study procedures were conducted. The trial was pre-registered on ClinicalTrials.gov with the identifier: NCT01965925.

This was a blinded, placebo-controlled study with a flexible dosing schedule. Patients were screened and, if deemed eligible, randomized on a 2:1 ratio such that two participants were assigned active drug (modafinil) for each participant assigned placebo. Dosing started at 100 mg/day qAM at baseline. If tolerated, the dose was increased to 200 mg/day qAM. Participants were instructed to take the drug upon waking, with no adjustment in sleep schedule. The visit schedule included weekly, in-person visits to track side effects and symptom ratings. Neurocognitive functioning was assessed by trained study staff at baseline and again at weeks 4 and 8 of the study.

### 2.1. Participants

Patients were recruited from a wide array of referral sources including: treatment centers at Mount Sinai Hospital (the Psychiatry Outpatient Clinic and the Mood and Anxiety Disorders Program); other research studies within the Mood and Anxiety Disorders Program; Mount Sinai Hospital system clinicians and outside affiliates such as Elmhurst Hospital in Queens; self-referrals from media advertisements; and referrals from consumer advocacy groups specializing in severe, treatment resistant mood disorders, including NAMI and the Mood Disorders Support Group (MDSG) of New York. All participants were recruited between March 2014 and November 2017.

Inclusion criteria were as follows: age between 18 and 65; DSM–IV BD I or II diagnosis; affective stability, defined by a Clinician-Administered Rating Scale for Mania (CARS-M) (18) rating of ≤8 and a Hamilton Rating Scale for Depression (HRSD) rating of ≤16 at screening and at the baseline visit. In this study, we did not require remission, rather we selected affective stability criteria that would enhance feasibility of recruitment, reduce chances of concerning side effects, and target aspects of the illness that are unmet needs (subthreshold depression, sleep problems, and persistent cognitive impairment). Thus, we employed a more liberal approach by allowing for HRSD ≤16. We were intentional in allowing subthreshold levels of depression as many if not most BD patients do not achieve a pure euthymia; however, due to the potential risk for treatment associated emergent mania, we were stricter on the CARS-M cutoffs. In addition we required clinically-acceptable, stably-dosed, mood stabilizing medication regimen for ≥1 month prior to enrollment, with no medication changes planned over the 8-week study period; objective evidence of either a subjective sleep quality complaint (Pittsburgh Sleep Quality Index total score > 5) and/or evidence of clinically-significant cognitive impairment at screening. Cognitive impairment at screening was determined using a short battery of tests not included in the MATRICS Consensus Cognitive Battery (MCCB) so as not to expose subjects to these tasks before baseline. Specifically, Trails B, Wechsler Adult Intelligence Scale (WAIS-IV) Digit Symbol; WAIS-IV Digit Span Forward and Backward; Wisconsin Card Sorting Test (WCST); and the California Verbal Learning Test (CVLT) were administered as a pre-screen for eligibility. Clinically-significant impairment was defined as scoring ≥1 standard deviation below normative means on at least one of these measures. An impairment of this degree is typically considered clinically relevant.

Exclusion criteria were as follows: history of CNS trauma, neurological disorder, ADHD, or learning disability; positive urine toxicology or DSM-IV diagnosis of substance abuse/dependence within the past 3 months; active, unstable medical problem that may interfere with sleep or cognition; history of substance-induced mania; recent history of rapid-cycling; score of 2 or greater on the decreased need for sleep item from the CARS-M (excludes insomnia-based sleep difficulties but addresses reduced sleep while still feeling well-rested); taking any drug known to interact with modafinil; more than 3 psychotropic medications; abnormal lab or ECG result at screening; significant suicidal ideation at baseline (HRSD item 3 > 2) or at risk for suicidal behavior based on clinical judgment; participation in any other investigational cognitive enhancement study within 30 days; pregnant or breast feeding; and treated with electroconvulsive therapy (ECT) within the last 12 months.

Practical and ethical considerations prevented exclusive focus on medication-free BD patients. However, we limited participation to individuals not taking any medications with known adverse cognitive effects (e.g., topiramate, tricyclics, and anticholinergics), agents that may enhance cognition (e.g., amphetamine and dopamine agonists), or benzodiazepines (e.g., lorazepam) for sleep (due to known effects on daytime fatigue and cognition). All other medications were recorded daily to assess changes of use in PRN agents while taking modafinil. Although some standard treatments for BD (e.g., lithium) may influence cognition, it was impractical to exclude these medications given their widespread use in BD.

### 2.2. Measures – safety

Safety was assessed weekly through clinician-administered and self-report measures, in addition to laboratory measures. Clinician administered self-report measures administered to ascertain safety at each visit included a side effects checklist (19), the Beck Scale for Suicidal Ideation (20), and the Columbia-Suicide Severity Rating Scale (21). Blood pressure was taken at every visit. At baseline and week 8, additional laboratory tests were conducted, including: electrocardiogram, liver function tests, chemistry panel, complete blood count, and urinalysis.

To further evaluate safety, clinical symptoms were also assessed at baseline and each weekly visit. At screening, diagnosis were made using the Structured Clinical Interview for the DSM-IV (22). Weekly clinical measures included the Hamilton Depression Rating Scale [HDRS; (23)], a 24-item, clinician-administered measure of depression severity over the past several days; and the Clinician-Administered Rating Scale for Mania [CARS-M; (18)], a 15-item, clinician-rated measure of severity of symptoms of mania and psychosis over the past 7 days.

### 2.3. Measures – clinical outcomes

Sleep quality and daytime wakefulness were measured at baseline and each weekly visit using the Pittsburgh Sleep Quality Index (PSQI) and Epworth Sleepiness Scale (ESS), respectively. The PSQI (24) is a self-report instrument measuring the quality and patterns of sleep in adults. It differentiates “poor” from “good” sleep in 7 areas: subjective sleep quality, latency, sleep duration, habitual sleep efficiency, sleep disturbances, use of sleeping medication, and daytime dysfunction. The Epworth Sleepiness Scale [ESS (25)] is a self-report questionnaire that measures daytime sleepiness. Subjects rate the probability of falling asleep on a scale of increasing probability from 0 to 3 in eight different situations. For both the PSQI and the ESS, higher scores indicate more impairment.

Cognitive functioning was measured at baseline, week 4, and week 8 using the MATRICS Consensus Cognitive Battery [MCCB; (26)]. All data collection and test administration were overseen by a neuropsychologist. MCCB domains include: Speed of Processing; Attention/Vigilance; Working Memory; Verbal Learning; Visual Learning; Reasoning & Problem-Solving; and Social Cognition and the battery takes approximately 80 minutes to complete. Alternate forms for the MCCB were administered at follow-up visits to minimize practice effects. The primary cognitive functioning outcome was the MCCB composite score, which represents a global measure of cognition and is the recommended outcome measure for inclusion in efficacy analyses (27). Past studies show deficits on this battery in euthymic BD patients supporting its use (28).

### 2.4. Statistical analyses

The results reported here are largely descriptive for safety and feasibility outcomes. For ease of interpretation, composite and subscale scores on the MCCB were converted to standard scores utilizing the MCCB normative scoring program, which are age- and sex-corrected and reported on a t-score scale (Mean = 50, SD = 10).

While this was a pilot study and underpowered for inferential statistics, we used mixed effects models to offer a *preliminary* assessment of efficacy on primary outcomes. All mixed effects models included the fixed predictors of treatment group (modafinil versus placebo), week in study (time; linear term), and the interaction of treatment group and time. The random effect was subjects nested in treatment group. Model residuals were examined for conformance to model assumptions.

## 3. Results

### 3.1. Feasibility

**Figure 1:**
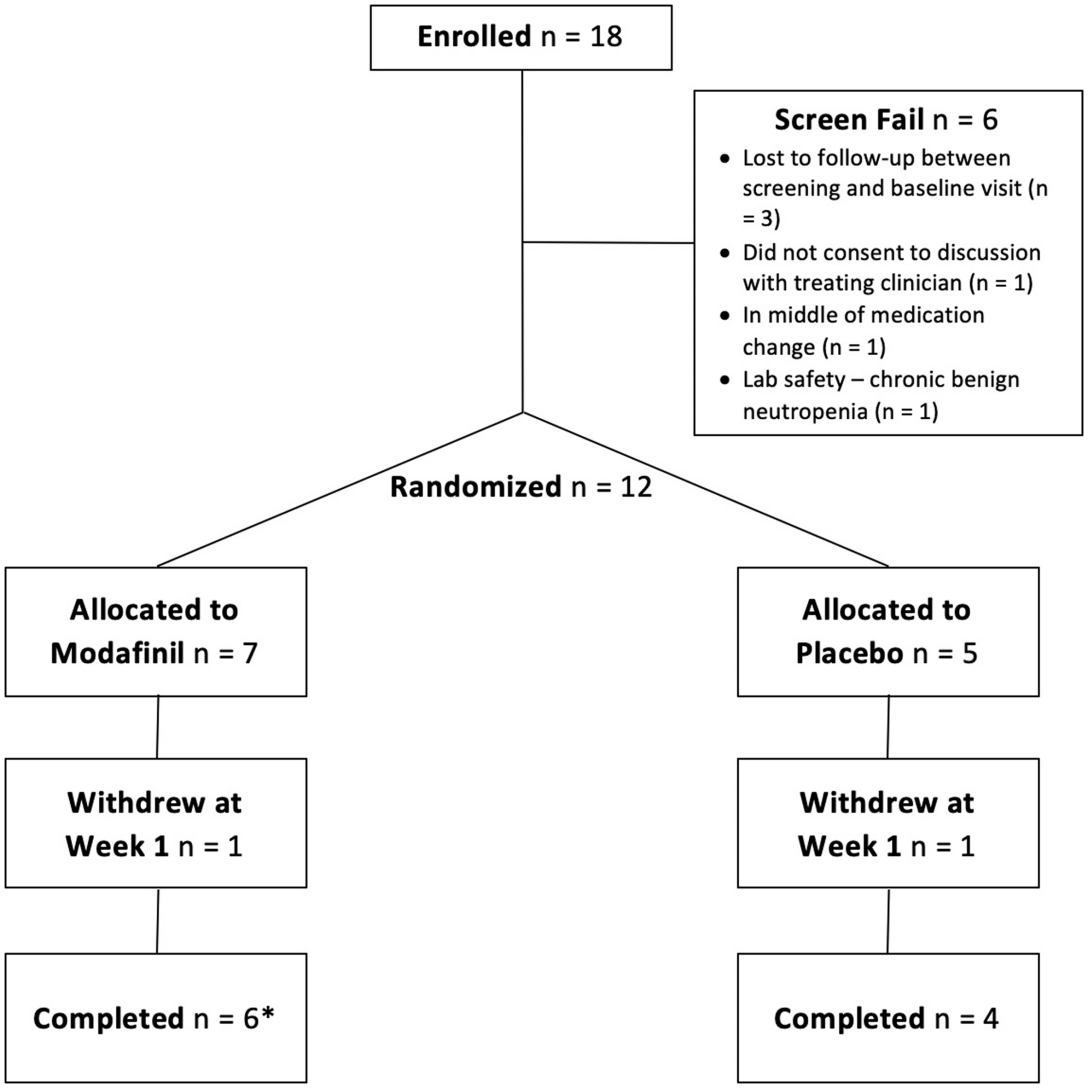
Consort diagram. One participant in the modafinil group withdrew at week 7 but is still included as a completer because she came in for her follow-up visit.

Recruitment was difficult and we had to stop the trial short of our intended sample size goal, but this was not necessarily unexpected as inclusion/exclusion criteria for clinical trials are often limiting. A total of 18 patients were consented to the study with 6 screen fails (see Figure 1). One patient in the modafinil group dropped out at week 1 owing to side effects (see below). One patient in the placebo group dropped out at week 1 due to the belief that she was assigned placebo and wanting to seek an off-label modafinil prescription.

### 3.2. Demographics

The final sample consisted of 10 completers. Demographics and baseline clinical characteristics for the completers are summarized in Table 1. The individual who dropped out of the modafinil group was a 30-year-old, White, non-Hispanic male with a diagnosis of BD I. The individual who dropped out of the placebo group was a 49-year-old, White, Hispanic, female with a diagnosis of BD I. Given low numbers, sensitivity analyses were not permitted. A table of baseline medications for all patients enrolled can be found in Supplementary material.

**Table 1:** Baseline characteristics by treatment group for completers.

	Modafinil	Placebo	Total
	Number (%)		
Female sex	4 (66.66%)	3 (75.00%)	7 (70.00%)
BD I diagnosis	6 (100.00%)	3 (75.00%)	9 (90.00%)
Race – White	4 (66.66%)	1 (25.00%)	5 (50.00%)
Race – Black	2 (33.33%)	3 (75.00%)	5 (50.00%)
Ethnicity – Hispanic	0 (0%)	1 (25.00%)	1 (10.00%)
	Mean (SD)		
Age	51.33 (11.15)	44.00 (13.29)	48.40 (11.93)
Years of education	15.00 (2.10)	14.50 (1.92)	14.80 (1.93)
Hamilton Depression Rating Scale (HDRS)	6.67 (3.08)	7.75 (4.35)	7.10 (3.45)
Clinician-Administered Rating Scale for Mania (CARS-M)	2.83 (3.06)	0.75 (0.96)	2.00 (2.58)
Composite cognitive functioning	42.17 (14.43)	40.25 (11.44)	41.40 (12.66)
Speed of processing	45.00 (15.28)	53.00 (12.03)	48.20 (13.97)
Attention/vigilance	45.00 (10.90)	36.00 (15.19)	41.40 (12.83)
Working memory	39.33 (16.13)	43.00 (8.37)	40.80 (13.10)
Verbal learning	46.50 (8.36)	43.25 (7.81)	45.20 (7.87)
Visual learning	47.00 (13.70)	45.00 (11.17)	46.20 (12.12)
Reasoning and problem solving	46.00 (10.73)	41.75 (5.12)	44.30 (8.81)
Social cognition	47.00 (1.90)	46.25 (12.04)	46.70 (7.10)
Pittsburgh Sleep Quality Index (PSQI)	11.00 (4.52)	11.50 (3.11)	11.20 (3.82)
Epworth Sleepiness Scale (ESS)	6.33 (6.25)	13.75 (3.50)	9.30 (6.36)

### 3.3. Safety

There were *no serious adverse events* reported in the conduct of this trial. Additionally, laboratory results from weekly (i.e., vitals) and the week 8 (i.e., electrocardiogram, liver function tests, chemistry panel, complete blood count, and urinalysis) visits did not indicate any clinically significant changes. Finally, all completers assigned to receive modafinil tolerated the maximum per protocol dose of 200 mg.

There was one patient assigned to the active treatment group who dropped out of the study at week 1 owing to subjective experience of decreased concentration and energy. Additionally, there was one patient assigned to the active treatment group whose CARS-M score indicated hypomania at week 7, therefore, by protocol, treatment was discontinued. This was a 44-year-old, White, non-Hispanic female with a diagnosis of BD I. Hypomania was resolved by the time she was seen at her next follow-up visit one week later.

Adverse events assessed via the side effects checklist and measures of suicidal ideation and behavior are detailed in Table 2. In the modafinil group, *newly* emergent side effects (change since baseline report) were: heart palpitations (1 new incidence); itching (1 new incidence); fatigue (1 new incidence); and decreased energy (2 new incidences). In the placebo group, *newly* emergent side effects were: frequent urination (1 new incidence); difficulty sleeping (1 new incidence); and poor concentration (1 new incidence). Also of note, all participants receiving modafinil treatment who reported poor concentration at baseline (*N* = 3) reported that this was resolved at week 8. Also of note, all participants receiving placebo who reported fatigue at baseline (*N* = 3) reported that this was resolved at week 8.

**Table 2:** Adverse events in completers at baseline and week 8.

Adverse event	Placebo group (*N* = 4)			Modafinil group (*N* = 6)		
	BL	Wk8	Notes	BL	Wk8	Notes
Diarrhea	0	0	–	0	0	–
Constipation	0	0	–	0	0	–
Dry mouth	0	0	–	2	0	Resolved since BL
Nausea/vomiting	1	0	Resolved since BL	0	0	–
Palpitations	0	0	–	1	2	*N* = 1 new since BL
Dizziness standing	0	0	–	0	0	–
Chest pain	0	0	–	0	0	–
Rash	0	0	–	0	0	–
Increased perspiration	0	0	–	0	0	–
Itching	0	0	–	0	1	*N* = 1 new since BL
Dry skin	1	0	Resolved since BL	1	0	Resolved since BL
Headache	2	0	Resolved since BL	1	0	Resolved since BL
Tremors	0	0	–	0	0	–
Poor coordination	0	0	–	1	0	Resolved since BL
Dizziness	0	0	–	0	0	–
Blurred vision	0	0	–	0	0	–
Ringing ears	0	0	–	0	0	–
Difficulty urinating	0	0	–	0	0	–
Painful urination	0	0	–	0	0	–
Frequent urination	0	1	New since BL	1	0	Resolved since BL
Menstrual irregularity	0	0	–	0	0	–
Difficulty sleeping	2	2	*N* = 1 resolved since BL *N* = 1 new since BL	3	2	*N* = 1 resolved since BL *N* = 0 new since BL
Sleeping too much	0	0	–	0	0	–
Loss of sexual desire	0	0	–	0	0	–
Trouble with orgasm	0	0	–	0	0	–
Trouble with erections	0	0	–	0	0	–
Anxiety	1	0	Resolved since BL	0	0	–
Poor concentration	2	1	*N* = 2 resolved since BL *N* = 1 new since BL	3	0	Resolved since BL
General malaise	0	0	–	0	0	–
Restlessness	1	0	Resolved since BL	0	0	–
Fatigue	3	0	Resolved since BL	2	2	*N* = 1 resolved since BL *N* = 1 new since BL
Decreased energy	3	1	*N* = 2 resolved since BL *N* = 0 new since BL	0	2	*N* = 2 new since BL
Other	0	0	–	0	0	–
Suicidal ideation	2	0	Resolved since BL	2	0	Resolved since BL
Suicidal behavior	1*	0	Resolved since BL	0	0	–
Suicide attempt	1	0	Resolved since BL	1	0	Resolved since BL

BL, baseline; * = participant reported non-suicidal self-injurious behavior at baseline.

### 3.4. Clinical outcomes

A summary of the tests of fixed effects for each of the 10 dependent variables evaluated is presented in Table 3. Graphs of mean scores over time for the 10 dependent variables can be found in Supplementary material.

**Table 3:** Mixed effects model results for main effects of treatment, time, and interaction terms.

Domain	Fixed effect	df^1^	df^2^	*F*	Reg. Coeff	Std. Error	*p*-value
Composite cognitive functioning	Treatment	1	8	0.04	−1.83	8.75	0.84
	**Time**	**1**	**18**	**26.08**	**0.90**	**0.18**	**<0.01**
	Treatment*Time	1	18	1.36	−0.33	0.29	0.26
Speed of processing	Treatment	1	8	0.42	6.26	9.68	0.54
	**Time**	**1**	**18**	**4.81**	**1.08**	**0.34**	**0.042**
	Treatment*Time	**1**	**18**	**3.40**	**−0.99**	**0.54**	**0.082**
Attention/vigilance	Treatment	1	8	1.32	−9.28	8.09	0.28
	**Time**	**1**	**18**	**6.76**	**0.67**	**0.33**	**0.018**
	Treatment*Time	1	18	0.00	0.021	0.52	0.97
Working memory	Treatment	1	8	0.15	3.50	8.89	0.70
	Time	1	18	2.35	0.96	0.40	0.14
	Treatment*Time	1	18	2.35	−0.96	0.62	0.14
Verbal learning	Treatment	1	8	0.21	−2.43	5.29	0.66
	**Time**	**1**	**18**	**3.08**	**0.083**	**0.34**	**0.096**
	Treatment*Time	**1**	**18**	**4.26**	**−1.11**	**0.54**	**0.054**
Visual learning	Treatment	1	8	0.07	−1.83	7.06	0.80
	Time	1	18	1.99	0.17	0.44	0.18
	Treatment*Time	1	18	0.87	0.65	0.69	0.36
Reasoning & problem solving	Treatment	1	8	0.31	−4.17	7.49	0.59
	**Time**	**1**	**18**	**17.89**	**0.88**	**0.27**	**<0.01**
	Treatment*Time	1	18	0.02	0.063	0.43	0.89
Social cognition	Treatment	1	8	0.07	1.28	4.68	0.79
	Time	1	18	1.38	0.40	0.38	0.26
	Treatment*Time	1	18	0.02	−0.083	0.60	0.89
PSQI	Treatment	1	8	0.95	1.71	1.75	0.36
	**Time**	**1**	**73**	**78.16**	**−0.35**	**0.093**	**<0.01**
	Treatment*Time	**1**	**73**	**17.35**	**−0.62**	**0.15**	**<0.01**
ESS	**Treatment**	**1**	**8**	**3.96**	**7.26**	**3.65**	**0.082**
	**Time**	**1**	**74**	**6.48**	**−0.12**	**0.14**	**0.013**
	Treatment*Time	1	74	2.15	−0.32	0.22	0.15

df^1^ = numerator degrees of freedom. df^2^ = denominator degrees of freedom. Reg. Coeff = partial regression coefficient estimate (i.e., effect size). For “Treatment” reg coeff represents the mean difference between treatment groups on the dependent variable with respect to the modafinil group. For “Time” reg coeff represents rate of change per week in study for the modafinil group. For “Treatment*Time” reg coeff represents the *difference* in rate of change between treatment groups with respect to the modafinil group as reference. Std. error = standard error of the partial regression coefficient estimate. Bold text indicates a significant or marginally significant effect.

Results for cognitive functioning outcomes showed several marginally significant effects of the interaction of treatment group and time. Specifically, for speed of processing the interaction term was marginally significant (*F*(1,18) = 3.40, *p* = 0.082). This finding suggested greater improvements in speed of processing over time in the modafinil group (slope = 1.08 units/week) than the placebo group (slope = 0.094 units/week). Similarly, for verbal learning the interaction term was marginally significant (*F*(1,18) = 4.26, *p* = 0.054). This finding also suggested greater improvements (or less decline) in verbal learning over time in the modafinil group (slope = 0.083 units/week) than the placebo group (slope = −1.03 units/week). Finally, as would be expected, there was a significant or marginally significant effect of time for several of the cognitive functioning variables, specifically improvements in composite cognitive functioning score, attention/vigilance, speed of processing, as well as reasoning and problem solving. These results likely reflect the well-documented practice effect with repeated exposure to cognitive testing.

Results for sleep–wake functioning outcomes also yielded some significant and marginally significant findings. Specifically, for PSQI, the interaction of treatment group and time was significant (*F*(1,63) = 5.40, *p* < 0.001). This finding suggested greater improvements in sleep quality over time in the placebo group (slope = −0.97 units/week) versus the modafinil group (slope = −0.35 units/week). For the ESS, there was a marginally significant main effect of treatment group, with the modafinil group having a lower mean across time (least squares mean = 5.32, standard error = 2.24) than the placebo group (least squares mean = 11.30, standard error = 2.74). This suggests that the modafinil group reported less daytime sleepiness than did the placebo group. Similarly, there was a significant effect of time on the PSQI and ESS, showing declines on these variables over time. These findings likely reflect treatment expectations on the part of participants.

## 4. Discussion

Preliminary evaluations of clinical efficacy revealed that the modafinil group showed greater improvement in specific cognitive domains (speed of processing and verbal learning) compared to the placebo group. Additionally, the modafinil group experienced reduced daytime sleepiness, while the placebo group showed greater improvement in sleep quality. These findings suggest a positive impact of modafinil on certain neurocognitive functioning and possibly daytime sleepiness, but that it may also have a negative effect on sleep quality. This pilot study provides preliminary evidence that modafinil is relatively safe when used adjunctively in patients with affectively-stable bipolar disorder, as no serious adverse events were noted. Findings did suggest that some patients may not tolerate modafinil well, as two patients assigned to modafinil discontinued the drug. However, rate of emergence of new side effects was reasonably comparable between the modafinil and placebo groups.

Modafinil has demonstrated its potential to enhance cognitive function in various populations, including individuals with major depressive disorder (MDD) and schizophrenia (16, 17). In a study involving remitted patients with MDD, it was found that a single 200 mg dose of modafinil resulted in improved episodic memory and working memory performance (17). However, the drug did not show notable benefits in planning or sustained attention. Moreover, based on a systematic review examining modafinil’s use in schizophrenia and related disorders, the drug demonstrated promising benefits in addressing cognitive, emotional, functional, and motor dysfunctions (29). Specifically, modafinil was observed to enhance various cognitive aspects, including working memory, short-term and long-term memory, attention, cognitive flexibility, and inhibitory control in individuals diagnosed with schizophrenia (29). Research in patients with a first episode psychosis revealed that modafinil exhibits positive effects on verbal and spatial working memory, as well as improvements in impulsivity-related tasks (30). However, the drug had no significant impact on sustained attention, attentional set-shifting, learning, or fluency. In modafinil studies involving patients with schizophrenia, reported side effects included itchiness, difficulty sleeping, and psychosis among others (30, 31).

Overall, existing literature indicates that modafinil shows promise as a therapeutic intervention for alleviating cognitive difficulties in patients with mood disorders. However, the specific impact of modafinil on cognition in individuals with BD remains largely unexplored and warrants further investigation. Most of the existing research has primarily focused on investigating the effect of modafinil on bipolar depression. A meta-analysis found that adjunctive treatment with modafinil/armodafinil was associated with a significant reduction in depressive symptoms in patients with bipolar depression (15). This paper was unable to perform a subgroup analysis for differences between bipolar subtypes (BD I versus BD II). One study found that patients with bipolar I disorder who received modafinil had significantly lower endpoint depressive symptoms compared to those with bipolar II disorder (32). The response rate to modafinil was also higher in the bipolar I group compared to the bipolar II group. These results suggest that modafinil may be more effective in reducing depressive symptoms in patients with bipolar I disorder compared to bipolar II disorder. Despite these promising results, additional research is needed to further explore the potential benefits of modafinil on cognitive functioning in patients with BD, including a more comprehensive examination of its effects on different bipolar subtypes.

The two instances of medication discontinuation in the modafinil group (owing to suspected side effects) compared to only one instance of discontinuation in the placebo group (not owing to suspected side effects) raise questions about tolerability. While two patients were a sizable percentage of the group assigned modafinil in this pilot study, with such a small comparison group we cannot rule out the possibility that side effects and symptoms reported were part of the normal course of disease in these patients. Although case studies have suggested that modafinil can produce manic symptoms in BD patients (33, 34), a recent meta-analysis suggested no difference in all-cause discontinuation or treatment-emergent mania in BD patients taking modafinil versus placebo for treatment of symptoms of depression (15). This rate of treatment emergent mania is similar to what is noted in other published antidepressant trials; however, we are very cautious in noting this as a meaningful finding given the very small denominator in this study. It should also be noted that results drawn from different settings report varying rates of treatment emergent mania (e.g., in a controlled trial, where a patient is seen weekly, a full switch to mania might be less likely than in regular clinical care) (35). Nevertheless, a key takeaway is that any off-label use of modafinil would require close monitoring by a prescriber.

The primary domain of interest for efficacy was cognition and findings suggested a positive signal on this outcome. Both processing speed and verbal learning showed marginally significant treatment group by time interactions, indicating a cognitive advantage in the modafinil group. The improvement noted on processing speed is in line with the primary mechanism of action of modafinil, as it is closely related to dopamine availability alongside the relative sensitivity of these measures to cognitive change. Interestingly, the treatment group by time interaction on verbal learning was driven in large part by a decline in performance on this domain in the placebo group. While this is unexpected, it is possible that it reflects the use of different/alternate forms on the verbal learning measure that may slightly differ in difficulty. There was minimal improvement in the modafinil group, but they did not show the same declining performance that was seen in the placebo group. Given our small sample size, these findings are far from conclusive. Nevertheless, they build a case for further investigation of the possible utility of modafinil in improving cognitive functioning in BD.

Findings with regard to daytime sleepiness and sleep quality were mixed. Specifically, one participant discontinued modafinil at week 1 owing to fatigue, two participants taking modafinil reported newly decreased energy at week 8, and sleep quality improved significantly more over time in the placebo group than the modafinil group. Taken together these findings suggest that modafinil may have a negative impact on sleep quality and energy. This conclusion is complicated, however, by our finding that patients taking modafinil reported less daytime sleepiness. It is possible that this finding is an artifact of random differences in baseline values across the modafinil and placebo groups. It is also possible that modafinil acts to decrease daytime sleepiness (expected) but to the detriment of nighttime sleep quality. Specifically, we speculate that sleep quality was impaired in the patients taking modafinil due to a possible circadian shift – as supported by significantly “better” daytime wakefulness. Our a priori hypothesis was that this shift would result in *improvements* in sleep quality but the timing of the drug administration may have resulted in more durable effects on wakefulness than expected, resulting in impairments in sleep. Future studies in this space should consider timing of dosage to address this issue and might also integrate wearable sleep monitoring to capture more systematic and objective markers of day-to-day sleep–wake functioning.

Our study had several limitations. First, it was designed as a pilot trial with the primary objective of exploring the feasibility, safety, and preliminarily efficacy of modafinil. Consequently, this study is underpowered for inferential statistics to determine treatment effects. With a larger sample size some of the differences observed may have been significant. Effect sizes indicate that a well-powered study might indeed be warranted. Second, small sample size studies are susceptible to inadvertent imbalances (despite randomization) between treatment groups at baseline on primary outcomes. This was most notable in the case of ESS, where the baseline score was substantially higher (worse) in the placebo group than in the modafinil group. Because of the small sample size in this pilot study, covarying baseline values in the mixed effects models would have resulted in very under-powered significance tests. The small sample size also limits the generalizability of the findings and the study’s design does not allow for a clear determination of whether the observed effects are due to modafinil itself or to other factors such as the mood stabilizers that the patients were also taking. Third, some of our eligibility criteria, for example, including participants with either poor sleep quality *and/or* clinically significant cognitive impairment at screening, may have increased the heterogeneity of the sample enough to weaken the potential signals observed on primary clinical outcomes.

Despite these limitations, this study provides important early data on the use of modafinil to improve neurocognitive and sleep–wake functioning in affectively-stable BD patients. Findings suggest that application of modafinil to improve inter-episode functioning may be promising when closely monitored by a prescriber – an important focus for treating BD patients to full recovery and optimizing quality of life. Further evaluation with a larger sample size is required for clearer conclusions. Furthermore, opportunities for objective monitoring of sleep–wake functioning using wearables and attention to profiles of patients who respond well versus poorly to modafinil will be essential to future clinical utility of findings from a larger trial.

## Data availability statement

The raw data supporting the conclusions of this article will be made available by inquiry to KEB, without undue reservation.

## Ethics statement

This human research study was approved by the Institutional Review Board (IRB) at the Icahn School of Medicine at Mount Sinai. The study was conducted in accordance with the local legislation and institutional requirements. The participants provided their written informed consent to participate in this study.

## Author contributions

KEB was primarily responsible for study design. KEB, MP-R, EL, and MS were key to data acquisition and safety monitoring, whereas JML, JL, and CKP were primarily responsible for planning and conducting data analysis. JML and MM were primarily responsible for drafting the manuscript. CKP, JL, MP-R, EL, MS, and KEB were involved in interpretation of findings and critical revision of the manuscript. All authors contributed to the article and approved the submitted version.

## Funding

This study was supported by a grant from the National Institute of Mental Health (NIMH) to KEB (R34 MH101267). Additionally, JML’s time was supported by a Career Development Award from NIMH (K23 MH120324).

## Conflict of interest

KEB serves on the steering committee for the non-profit Breakthrough Discoveries for thriving with Bipolar Disorder (BD^2) and receives grant funding and honoraria in this capacity.

The remaining authors declare that the research was conducted in the absence of any commercial or financial relationships that could be construed as a potential conflict of interest.

## Publisher’s note

All claims expressed in this article are solely those of the authors and do not necessarily represent those of their affiliated organizations, or those of the publisher, the editors and the reviewers. Any product that may be evaluated in this article, or claim that may be made by its manufacturer, is not guaranteed or endorsed by the publisher.
