# Supplementary material for: Modafinil’s effects on cognition and sleep quality in affectively-stable patients with bipolar disorder: a pilot study
Source: Front Psychiatry. 2023 Sep 4;14:1246149. doi: 10.3389/fpsyt.2023.1246149 (PMC10507316; doi:10.3389/fpsyt.2023.1246149)
Supplement: Supplementary file 1 [file Data_Sheet_1.pdf]

Appendix 1. Table of baseline medications for all participants enrolled

ID	Modafinil (Y/N)	Mood Stabilizer	Lithium	Antidepressant	Other Psychotropic	Other
1001	Y	Quetiapine	-	Sertraline	-	Glyburide; HCTZ; Naproxen
1003	N	Lamotrigine	-	-	Tramadol	Metformin; Colesevelam; Carvedilol; Atenolol
1004	Y	Lamotrigine	Y	-	-	Iron Tablets; Amlodipine/ Benazepril
1006	N	Aripiprazole	-	Sertraline	Clonazepam; Zolpidem	Atorvastatin; Ranitidine
1007	Y	-	Y	Duloxetine	Alprazolam	Enalapril; Crestor; Imitrex
1009	N	Valproic acid	-	-	-	Budesonide/ Formoterol
1010	Y	Lamotrigine	Y	-	-	-
1012	Y	Aripiprazole	-	Venlafaxine; Mirtazapine	Clonazepam	Levothyroxine; Pramipexole
1013	N	Lurasidone	-	Escitalopram	-	-
1014	Y	Lamotrigine	Y	-	-	-
1015	Y	Olanzapine	-	Fluoxetine	-	-
1016	Y	Lamotrigine	-	Sertraline	-	Premarin; Progesterone; Naratriptan; Lo Q10

Note. Participants 1014 and 1016 dropped out of the study at week 1 and participant 1004 was withdrawn from treatment per protocol at week 7 owing to hypomania.

Appendix 2. Mean scores over time for cognitive functioning variables with error bars

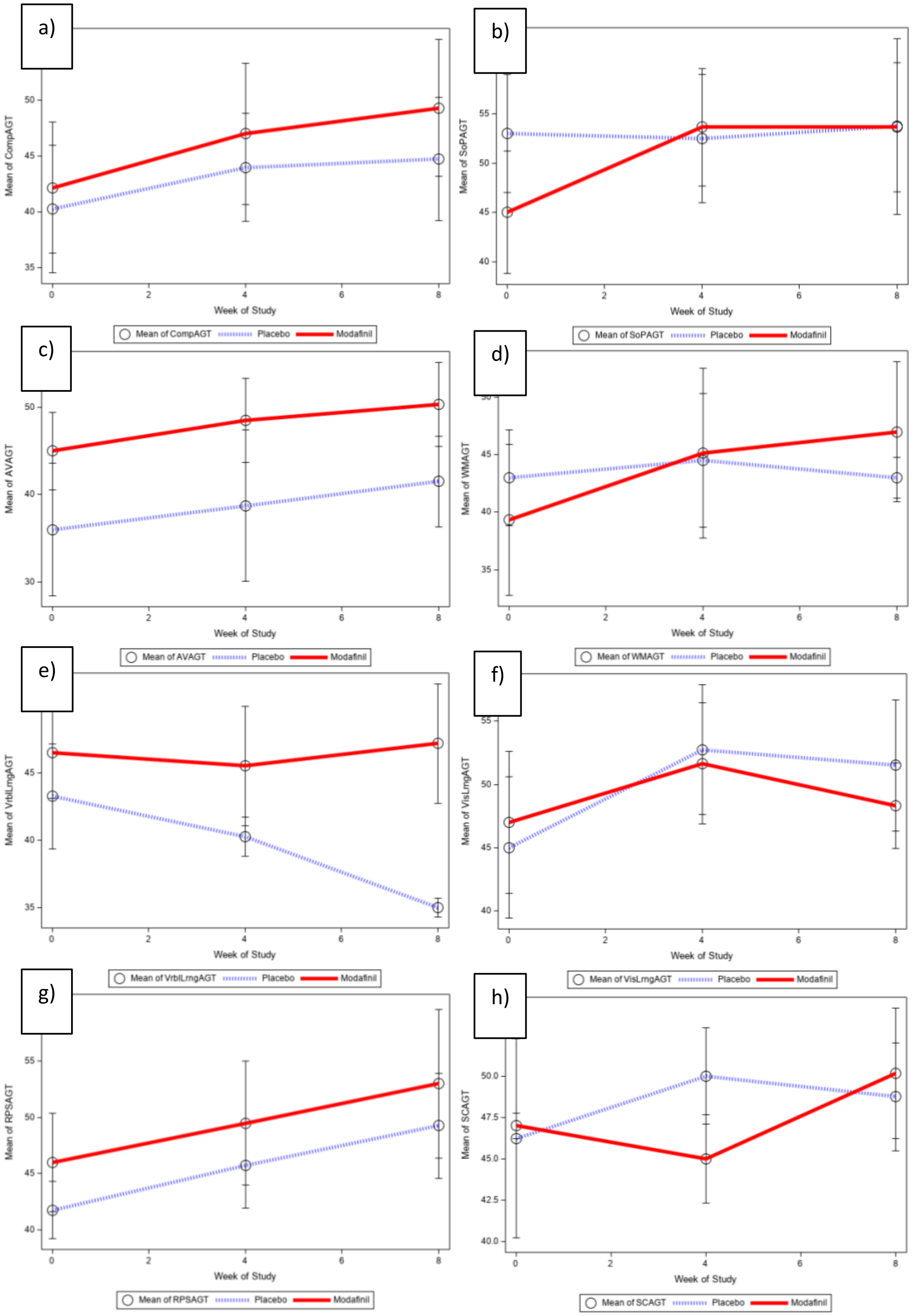

Note. CompAGT = composite cognitive functioning; SoPAGT = speed of processing; AVAGT = attention/vigilance; WMAGT = working memory; VrblLrngAGT = verbal learning; VisLrngAGT = visual learning; RPSAGT = reasoning and problem solving; SCAGT = social cognition

Appendix 3. Mean scores over time for sleep-wake functioning variables with error bars

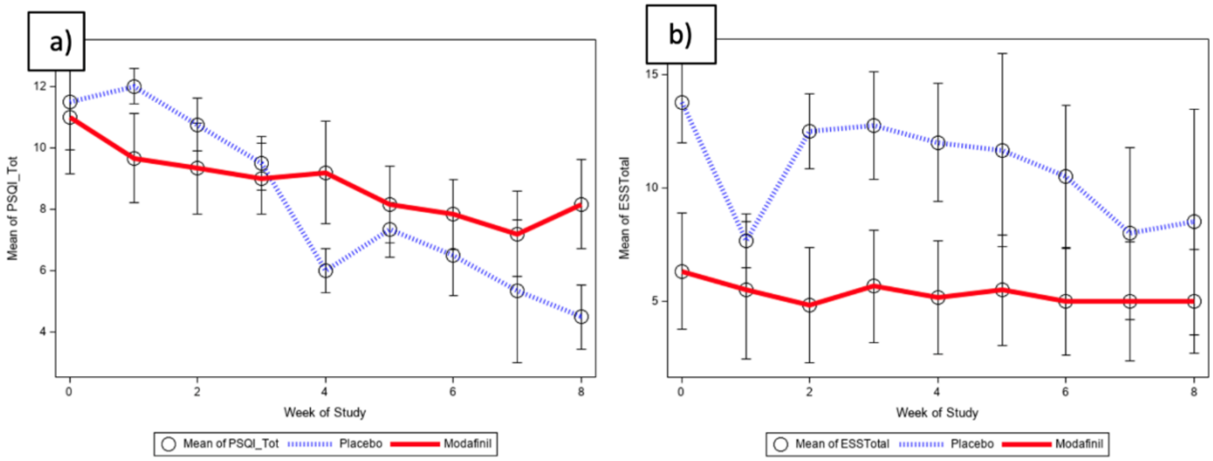

Note. PSQI_Tot = Total score on the Pittsburgh Sleep Quality Index (PSQI); ESSTotal = Total score on the Epworth Sleepiness Scale (ESS)
